# Sustained corticosterone rise in the prefrontal cortex is a key factor for chronic stress-induced working memory deficits in mice

**DOI:** 10.1016/j.ynstr.2019.100161

**Published:** 2019-04-04

**Authors:** Gaelle Dominguez, Nadia Henkous, Thomas Prevot, Vincent David, Jean-Louis Guillou, Catherine Belzung, Nicole Mons, Daniel Béracochéa

**Affiliations:** a Université de Bordeaux, CNRS UMR 5287, 33615, Pessac, France; b Université François Rabelais, Inserm U930, Parc Grandmont, 37200, Tours, France

**Keywords:** Stress, Prefrontal cortex, Hippocampus, Glucocorticoids, Working memory

## Abstract

Exposure to prolonged, unpredictable stress leads to glucocorticoids-mediated long-lasting neuroendocrine abnormalities associated with emotional and cognitive impairments. Excessive levels of serum glucocorticoids (cortisol in humans, corticosterone in rodents) contribute notably to deficits in working memory (WM), a task which heavily relies on functional interactions between the medial prefrontal cortex (PFC) and the dorsal hippocampus (dHPC). However, it is unknown whether stress-induced increases in plasma corticosterone mirror corticosterone levels in specific brain regions critical for WM. After a 6 week-*UCMS* exposure, C57BL/6 J male mice exhibited increased anxiety- and depressive-like behaviors when measured one week later and displayed WM impairments timely associated with increased plasma corticosterone response. In chronically stressed mice, basal phosphorylated/activated CREB (pCREB) was markedly increased in the PFC and the CA1 area of the dHPC and WM testing did not elicit any further increase in pCREB in the two regions. Using microdialysis samples from freely-moving mice, we found that WM testing co-occurred with a rapid and sustained increase in corticosterone response in the PFC while there was a late, non-significant rise of corticosterone in the dHPC. The results also show that non-stressed mice injected with corticosterone (2 mg/kg i.p.) before WM testing displayed behavioral and molecular alterations similar to those observed in stressed animals while a pre-WM testing metyrapone injection (35 mg/kg i.p.), a corticosterone synthesis inhibitor, prevented the effects of *UCMS* exposure. Overall, the abnormal regional increase of corticosterone concentrations mainly in the PFC emerges as a key factor of enduring WM dysfunctions in *UCMS*-treated animals.

## Highlights

• Unpredictable chronic stress (UCMS) produced protracted working memory deficits (WMD).
• WMD co-occurred with increased corticosterone and altered CREB activation in the PFC.
• Blockade of corticosterone synthesis by metyrapone prevented all the UCMS effects.
• Inversely, corticosterone injected in unstressed mice induced all the UCMS effects.

## Graphical abstract

**Figure:**
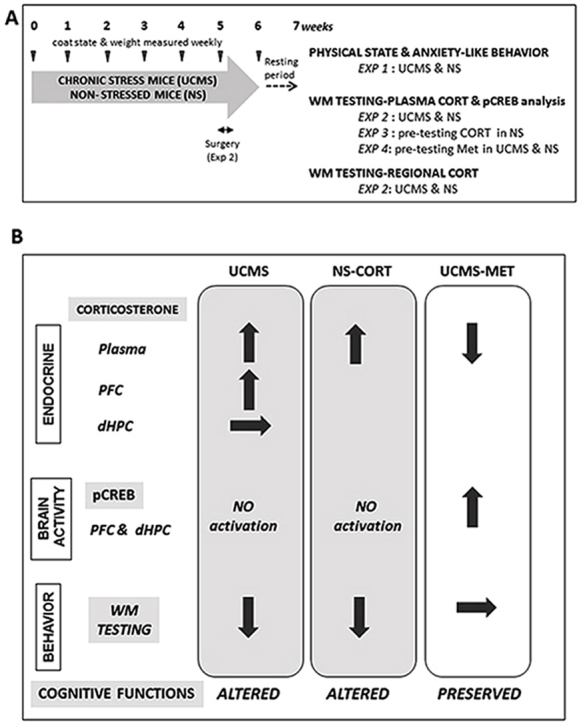

## 1 Introduction

Major depression, a pathology that can be triggered by chronic psychosocial stress in vulnerable subjects (Kendler et al., 2001), is characterized by mood disturbance, anhedonia, weight changes and sleep disturbance. Depression is also known to be associated with attention, learning and memory disturbances. Indeed, depressed patients show disrupted functional connectivity between the hippocampus (HPC) and the prefrontal cortex (PFC) (Geng et al., 2016).

At a neuroendocrine level, patients with severe depressive disorders are characterized by dysfunctional hypothalamic-pituitary-adrenal (HPA) axis (Nestler et al., 2002; Tanti and Belzung, 2013) and abnormal hypersecretion of glucocorticoids (GCs) hormones (corticosterone in rodents and cortisol in humans). The PFC and the HPC are particularly sensitive to the prolonged exposure to a high concentration of GCs and play an important role in mediating the effects of chronic stress on cognition. Studies using rodent models have shown that chronic stress and accompanying elevation of GCs dramatically impact the functioning of the PFC and the dHPC, especially through dendritic atrophy and/or remodeling and reduced synaptic connectivity (For review, Willner, 2016). Prolonged exposure to corticosterone also causes alterations of many different neurotransmitters systems or of their receptors density and affinity (Drevets, 2000; Yuen et al., 2012), as well as neurogenesis in the HPC (Tanti and Belzung, 2013). These structural and functional brain changes ultimately result in altered synaptic plasticity and cognitive impairments in HPC- and PFC-dependent memory tasks (McEwen, 2002; Sandi, 2004; Touyarot et al., 2004), including spatial working memory (WM) tasks. In humans, the pharmacological (hydrocortisone administration) or pathological (Cushing's disease) increases of cortisol can result in long-lasting spatial WM impairments (Lupien et al., 1999; Starkman et al., 2001; Patil et al., 2007; Luethi et al., 2009). This view is also supported by findings reporting that the administration of corticosterone or local infusion of GC receptor (GR) agonist mimics the stress-related WM impairment in rodents (Roozendaal et al., 2004; Cerqueira et al., 2005; Arnsten, 2009).

However, it is unclear whether the elevation of plasma corticosterone in response to a stressor in rodent models of depression accurately reflects enhanced corticosterone concentrations in specific brain regions. Some studies already reported that plasma and brain corticosterone responses display distinct temporal dynamics after exposure to stress (Droste et al., 2008; Heinzmann et al., 2010). For example, stress resulting from prolonged alcohol consumption and withdrawal elicited abnormal increases in corticosterone concentrations specifically in the prelimbic (PL) region of the PFC and the dorsal HPC (dHPC), while plasma concentrations remained unchanged (Little et al., 2008; Dominguez et al., 2017).

Studies in rodents have reported brain region-specific differences in translocation patterns of GC receptors in response to behavioral stress or corticosterone administration (Sarabdjitsingh et al., 2009; Caudal et al., 2014). Previously, we evidenced that acute footshock stress induces persistent and distinct corticosterone rises along the dorsal and ventral axis of the HPC at a time at which plasma levels were already returned to baseline (Dorey et al., 2012). From these findings, we hypothesized that such enduring region-specific changes of corticosterone within the PFC and the dHPC might play an important role in the effects of chronic stress exposure on cognition and emotional changes.

To that aim, this study attempted to determine the temporal dynamics of corticosterone patterns in the PL subregion of the PFC and dHPC in relation with chronic stress-induced behavioral and molecular alterations. To address this issue, we used the unpredictable chronic mild stress paradigm (*UCMS*), which induces behavioral, cognitive and biological alterations, paralleling the human pathological condition (Belzung and Surget, 2008).Using intracerebral microdialysis, we first assessed whether chronically-stressed mice displayed region-specific changes of corticosterone responses during WM testing in the PFC and the dHPC, the key regions for HPA axis GC feedback. We also examined whether chronic stress affected the activation/phosphorylation of the transcription factor cAMP-response element binding (CREB) in the PFC and in the CA1 area of the dHPC 30min after the beginning of WM testing. Numerous studies have reported that an increase in activated/phosphorylated level of CREB (pCREB) is a key regulator in the formation, consolidation, and enhancement of memory (for review, Kida and Serita, 2014). Altered activation of the cAMP-Protein kinase A (PKA)-CREB signaling cascade in specific brain regions has been implicated in behavioral models of anxiety and depression (Kuipers et al., 2006; Tardito et al., 2006). Accordingly, we have recently provided evidence that one route by which persistent rise of prefrontal corticosterone concentration induces anxiety-like behaviors and WM deficits long after the cessation of prolonged alcohol consumption may be through sustained decreased activity of the PKA-CREB signaling cascade (Dominguez et al., 2017). Given our previous data, we further intended in the present paper to examine the effects of a single injection of corticosterone administrated to non-stressed mice 30min before WM testing or, inversely, of a single, pre-testing injection of metyrapone (an inhibitor of corticosterone synthesis) administrated to chronically stressed mice on WM performance and region-specific pCREB expression. Our data indicate that the PFC and the dHPC differ in their sensitivity to chronic stress exposure and that abnormal sustained rise of corticosterone specifically in the PFC emerges as a key factor of chronic stress-induced behavioral deficits.

## 2 Materials and methods

### 2.1 Animals

**Fig. 1:**
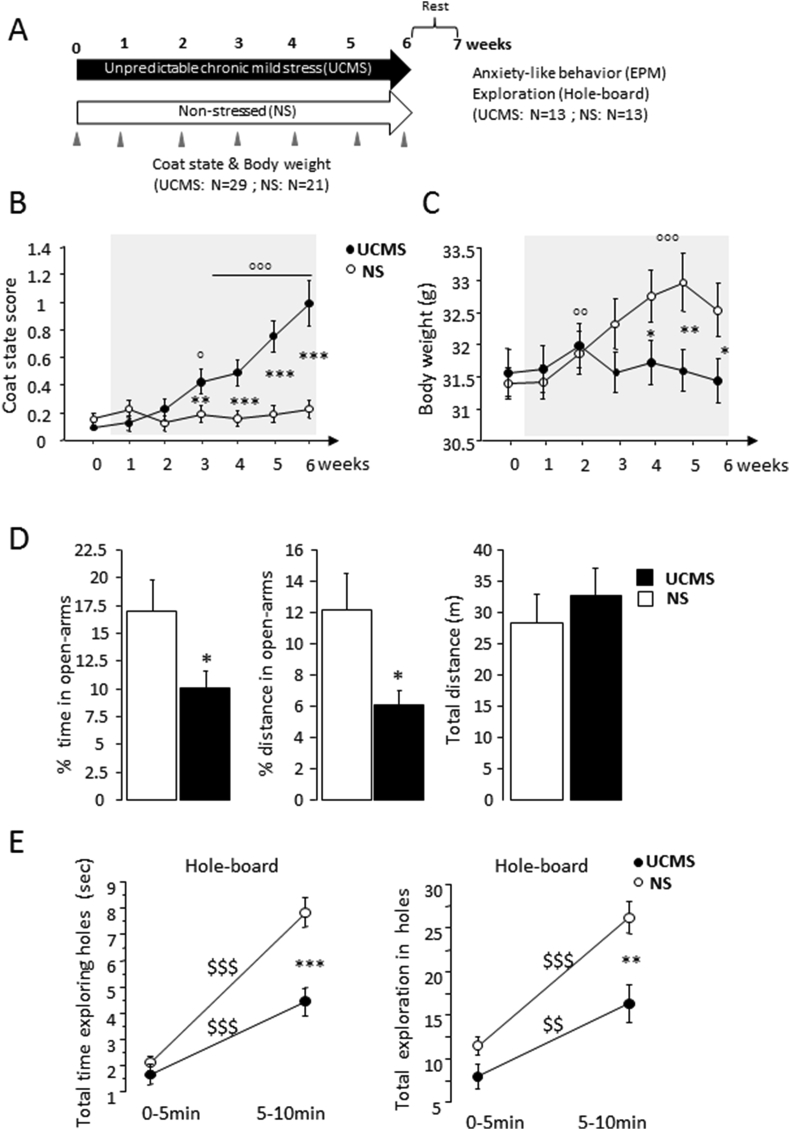
**The effects of *UCMS* exposure on physical state and anxiety-like behaviors. (A)** Schematic representation of the experimental procedures. Mice were randomly assigned into two groups: *UCMS* (N = 29) or non-stressed mice (NS, N = 21). Measurements of the coat state scores and body weight were assessed weekly (arrows). After a 1-week resting period, anxiety-like behavior in the elevated plus maze (EPM) and exploration in the hole-board apparatus were conducted in a cohort of mice from the *UCMS* and NS groups (N = 13 for each group). *UCMS* mice displayed significant deterioration of the coat state score **(B)** and body weight **(C)** after 3–4 weeks of stress condition, compared with NS mice. **(D)** In the EPM test, *UCMS* mice displayed decreased percentage of time (D_left_), and distance (D_middle_) in the extremities of open arms compared to NS mice. The total distance spent in the maze (D_right_) did not differ between the groups. **(E)** In the hole-board test, *UCMS* produced significant decreases of the total time (in sec) exploring the holes (**E**_**left**_) and number of total holes visited (**E**_**righ**t_) during the 5–10min period. Bars represent means ± SEM. *P < 0.05, **P < 0.01 and ***P < 0.001 relative to NS. °P < 0.05; P < 0.01 and P < 0.01 relative to weeks 0–1. ^$$^: p < 0.01 and ^$$$^: p < 0.001 relative to 0–5min period.

Male C57BL/6 J mice (24 weeks old; Charles River, L'Arbresle, France) were housed by groups of 10 mice until they were 9 months-old. All mice were maintained at 22 ± 1 °C, under a 12:12 light-dark cycle (lights on at 7:00 a.m.). They were provided with food and water ad libitum. During the whole *UCMS* period, mice were randomly assigned into 2 groups: the *UCMS* mice (N = 65) were kept alone (cage size: 27 × 10 × 12 cm) whereas mice that did not undergo *UCMS* exposure (non-stressed, N = 82) were kept 4–5 per cage (cage size: 30 × 20 × 12 cm) and housed in a separate room having no contact with the stressed mice (Fig. 1A). All experimental procedures were performed between 8:00 and 12:00 a.m. to avoid any side effects of the circadian rhythm on GCs levels (Ottenweller et al., 1979; Coleman et al., 2016). All procedures were approved by the local Ethics Committee for Animal Experiments (N° 501-20-89) and were performed in accordance with the European Communities Council Directive of 1st February 2013 (2010/63/EU).

### 2.2 *UCMS* paradigm

The *UCMS* procedure was conducted during 6 consecutive weeks as previously described (Belzung and Surget, 2008). The stressors consisted in alterations of the bedding (repeated changes, sawdust removal, damp sawdust, placement in a cage with water), cage-tilting, switching cages), and restraint stress. Body weight and coat state were assessed weekly. Coat state index, which is an index of general physical state, was evaluated on seven different body parts as previously described (Griebel et al., 2002; Surget et al., 2008). Briefly, the state of their coat was evaluated on 7 body parts (head, neck, back, ventral coat, hind paws, forepaws and tail). All parts were scored as a function of the deterioration state (0 for a good state, 0.5 for a mild deterioration and 1 for an important deterioration).The final score was obtained by adding the scores for each body part and dividing by the total number of body parts. After the 6 week-*UCMS* exposure, the *UCMS* and NS mice were handled daily (5min per day) throughout the one week rest period to minimize the effects of non-specific stress.

### 2.3 Behavioral tests

#### 2.3.1 Elevated plus maze

The elevated plus-maze task was performed to assess anxiety-like behavior. Mice were placed in the centre intersection for 30s in a cylinder to allow first orientation in the maze at random. Then, they were allowed to freely explore the maze for 10min. Animal behavior was recorded using an automated tracking system (Videotrack, Champagne au Mont d’Or, France), allowing measurements of the time and distance (m) spent by area. An entry was counted only when a mouse entered an arm with all four paws. The time or distance spent in the open arms divided by the total time or distance spent in all arms of the maze was used to measure anxiety-like behavior during the first and second 5-min blocks. The smaller are the ratios, the more “anxious” is the mouse. After each trial, mice were placed back in their home cage with littermates.

#### 2.3.2 Hole board exploration

Insofar as the alternation behavior used to evaluate WM is based on innate exploratory tendency, we evaluated the spontaneous exploratory activity in a hole-board apparatus. Each mouse was allowed to explore the hole-board apparatus (45 × 45 × 30 cm) for 10 min. The hole-board apparatus was made of white acrylic plate with8holes (3 cm in diameter, arranged in 3 × 3). Photoelectric cells were inserted in each hole, allowing automatic recordings of the number of head-dips performed in each hole.

#### 2.3.3 Working memory task

**Fig. 2:**
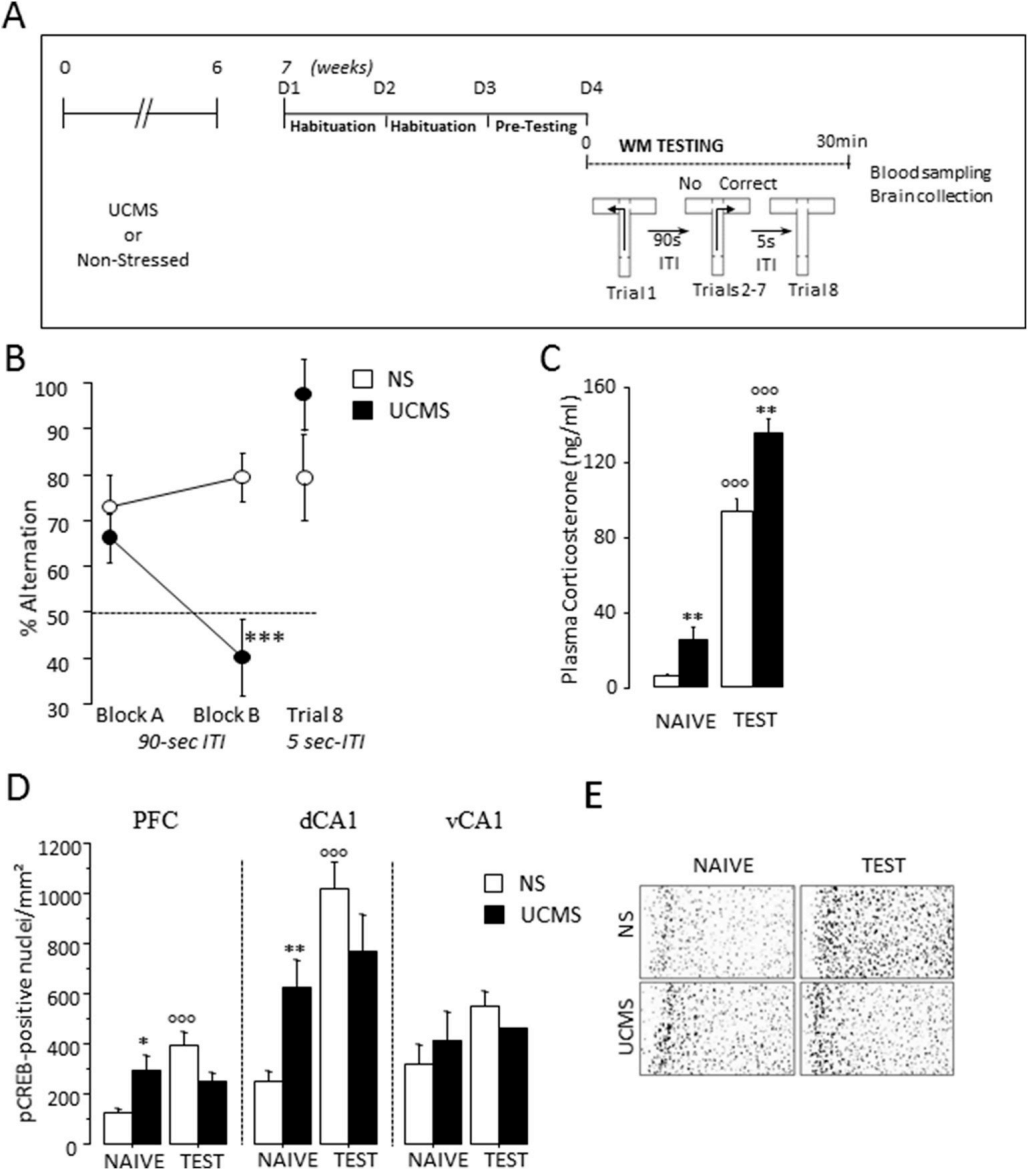
***UCMS* leads to WM deficits associated with increased plasma corticosterone response and decreased pCREB immunoreactivity. (A)** Experimental design: plasma corticosterone and number of pCREB-positive neurons were measured in the remaining *UCMS* and non-stressed (NS) mice sacrificed 30min after the beginning of WM testing (*UCMS*: N = 10*; NS*: N = 8) in the T-maze alternation task. **(B)**; ***Left:*** Mean percentage of alternation rates were measured in Block A (Trials 2–4) and Block B (Trials 5–7) with a 90-sec inter-trial interval (ITI). ***Right:***trial 8 had a shorter 5-sec ITI. **(C)***UCMS* exposure significantly enhanced plasma corticosterone (ng/ml) under baseline condition (*NAIVE)* and after WM (*TEST*). **(D)** The numbers of positive pCREB nuclei/mm^2^were measured in the PFC, the dCA1 and the vCA1. In the *NAÏVE* groups, the *UCMS* mice had significantly higher pCREB-positive neurons in the PFC and the dCA1 compared to NS mice. WM testing induced significantly higher pCREB-positive neurons in both structures of the *NS*, but not of the *UCMS* mice. No effect of TEST nor *UCMS* was found in the vCA1. **(E)** Representative photomicrographs showing pCREB immunoreactivity in the PFC of NS *(top)* and *UCMS (bottom)* mice from the *NAIVE (left)* and *TEST (right)* groups. Bars represent means ± SEM.*P < 0.05, **P < 0.01 and ***P < 0.001 relative to the *NS*. P < 0.001 relative to the *NAIVE*.

After a one-week rest period, *UCMS* and non-stressed mice were trained on a sequential alternation task over a series of successive trials in a T-maze (Fig. 2A). Repetitive testing constitutes a potent source of proactive interference. From trial to trial, accurate performance at a given trial (N) requires for subjects to be able to discriminate the specific target trial N-1 from the interfering trial N-2. The target information required for successful performance varies from trial to trial, so that the subject is not only required to keep temporarily in short-term memory specific information, but also to reset it over successive runs. The resetting mechanisms and cognitive flexibility required to alternate over successive runs are major components of WM processes. WM is a component of the sequential alternation task, since SA rates are dependent on the length of the intertrial delay interval (ITI), and/or the place of the trial in the series (Vandesquille et al., 2013).

Animals were first submitted to two free habituation sessions on two successive days (one session of 10min per day) in order to familiarize them to the apparatus and the allocentric spatial cues. During this phase, all doors of the T-maze were open and the mice were free to explore the apparatus. On the third day, the training phase consisted of seven successive trials separated by a 30-sec inter-trial interval (ITI). At each trial, the mouse was placed in the start box for 30-sec ITI before the door to the stem was opened. When the subject entered one of the goal arms, the door to that arm was closed and the choice was recorded. After a 30-sec confinement period into the chosen arm, the mouse was placed back in the start box for a new trial. To avoid olfactory cues in the apparatus, visible traces of urine and feces were washed with water.

**Test session**. Since no sequential alternation deficits were observed among groups in the training phase, mice were submitted 24-h later to the same procedure but with a 90-sec ITI. Lengthening the ITI increases delay-dependent interference over the series (Vandesquille et al., 2013). An alternation response was scored each time the subject entered the arm opposite to the one visited on the immediate preceding trial. Alternation rate was calculated taking into account the successive trials, and expressed in percentage relative to the maximal alternation rate of 100% (obtained when the subject never returned into the same arm over two consecutive trials). To dissociate memory deficit from an eventual progressive loss of motivation to alternate over the series, an 8th trial was added which was separated by a shorter ITI (5-sec) from the 7th one. Indeed, if alternation deficits depended on delay-dependent memory processes and not of motivation to alternate, then reducing the length of the ITI between the 7th and 8th trial should result in an increased alternation rate at the 8th trial.

### 2.4 Plasma and intracerebral corticosterone assays

#### 2.4.1 Plasma corticosterone

Blood samples were collected by sub-mandibular procedure with 25 gauge needles after anesthesia (Isoflurane^®^) in mice left undisturbed (baseline condition) or 30-min after the beginning of WM testing (Test condition). The blood was collected and serum was separated and stored at −80 °C until analysis.

#### 2.4.2 Intracerebral microdialysis

Mice were anesthetized with ketamine (100 mg/kg; body weight, i.p.) and xylasine (10 mg/kg; body weight, i.p.) (Bayer, Wuppertal, Germany) and implanted unilaterally with a guide-probe of microdialysis (CMA/7 Microdialysis, Sweden) above the PL (anteroposterior + 1.9 mm, lateral ± 0.3 mm and depth 1.2 mm; from Bregma) and the CA1 region of the dHPC (dCA1: anteroposterior -2mm, mediolateral ±1.4 mm, dorso-ventral −0.9 mm; from Bregma) (Paxinos and Franklin, 2001). The habituation and stabilization phases were performed as described in our earlier study (Dominguez et al., 2014; Dorey et al., 2012). The baseline dialysates were collected every 15-min before (60-min), during (30-min) and after (90-min) WM testing. During behavioral testing, the removable swivel bracket was placed above the maze allowing the mouse to move freely; then the swivel bracket was replaced above the dialysis bowl at the end of testing. All dialysates (collected between 8 and 12 a.m) were stored at −80 °C until corticosterone measurements. Plasma and dialysate corticosterone concentrations were quantified using a commercially Enzyme Immunoassay kit (Correlate-EIATM, Assay Designs, Ann Arbor, MI). The sensitivity of the assay was 0.08 nmol/L. Therefore, baseline sample concentrations were more than 10-fold superior than sensibility threshold.

For each group, the effects of *UCMS* and testing were analyzed as % variations from baseline concentrations. This analysis allows to correct for imbalance between groups at baseline level. Percent variations from baseline concentrations are widely used and provide an immediate view of the main effects of treatments over time.

### 2.5 Immunohistochemistry

Immunohistochemical detection of the effects of *UCMS* and testing on pCREB immunoreactivity was performed as previously reported (Dominguez et al., 2016). Briefly, anesthetized mice were transcardially perfused with a fixative solution containing 4% paraformaldehyde in ice-cold phosphate buffer (PB; 0.1 M; pH 7.4). Brains were removed, post fixed overnight and sectioned (50 μm) on a Vibratome (Leica) and sections were stored at - 20 °C in a solution containing 30% ethylene glycol, 30% glycerol, 0.1 M PB until processed for immunohistochemistry. After incubation in blocking solution containing 8% goat serum and 0.05% Tween 20 (Sigma-Aldrich) in Tris buffer (TB; 0.1 M) for 1 h at room temperature (RT), free floating sections were incubated with rabbit primary polyclonal antibodies anti-phospho(ser133)-CREB (1:6000 in the blocking solution; Cell Signaling Technology, Beverly; USA) for 48 h at 4 °C. Sections were then rinsed in Tris buffer saline (TBS) and incubated for 2 h in blocking solution containing a biotinylated goat anti-rabbit IgG secondary antibody (1:2000, Jackson Immunoresearch) followed by an incubation with avidin-biotinylated horseradish peroxidase complex (Vectastain Elite kit, Vector Laboratories) for 2 h at RT. Sections were washed in TBS followed by TB, and the peroxidase reaction was visualized by using diaminobenzidinetetrahydrochloride and H_2_O_2_ (Sigma-Aldrich). Sections were mounted on gelatine-coated slides, dehydrated and cover slipped.

Images of the CREB-positive cells in the PL subregion of the PFC and the dHPC and the ventral HPC (vHPC) sections were acquired by using a 3CDD color video color Sony camera mounted on a BX-50 Olympus microscope. The quantification of positive nuclei was performed at 10× magnification. The pCREB-positive nuclei in the areas of interest were measured using our homemade ImageJ/Fiji developed by G. Courtand (INCIA, Bordeaux, France). Briefly, for each animal, regions of interest were delineated by an observer blind to experimental groups and labeled neurons were automatically counted given that they were above a threshold determined from each side of 3–4 sections. The data obtained in both sides of each section were pooled and expressed as mean number of pCREB immunopositive nuclei/mm^2^ in the PL subregion of the PFC, the dCA1 and the vCA1 according to Paxinos and Franklin atlas (2001).

### 2.6 Pharmacological treatments

Corticosterone (2 mg/kg; Sigma) and metyrapone (35 mg/kg; Tocris) were dissolved in 5% DMSO. The doses of metyrapone and corticosterone were chosen according to previous studies (Dominguez et al., 2017).The mice randomly received a single i.p. injection of drug or vehicle and performed WM testing 30min later. Then, 30min after the beginning of behavioral testing, blood was quickly collected (within 1min) and all *TEST* mice were sacrificed for brain collection. For measurements of baseline levels, mice randomly received a single i.p. injection of drug or vehicle and were sacrificed 1 h later. For the experiment 3 (Fig. 4A), the non-stressed mice were randomly assigned to naïve (CORT or VEH: N = 3 for each) or *TEST* (CORT: N = 7; VEH: N = 8) groups. For the experiment 4 (Fig. 5A), the *UCMS* and non-stressed mice were also assigned to naïve (MET or VEH: N = 4 for each) or *TEST* (non-stressed: VEH: N = 8 and MET: N = 8; *UCMS*: VEH: N = 7 and MET: N = 8) groups.

**Fig. 3:**
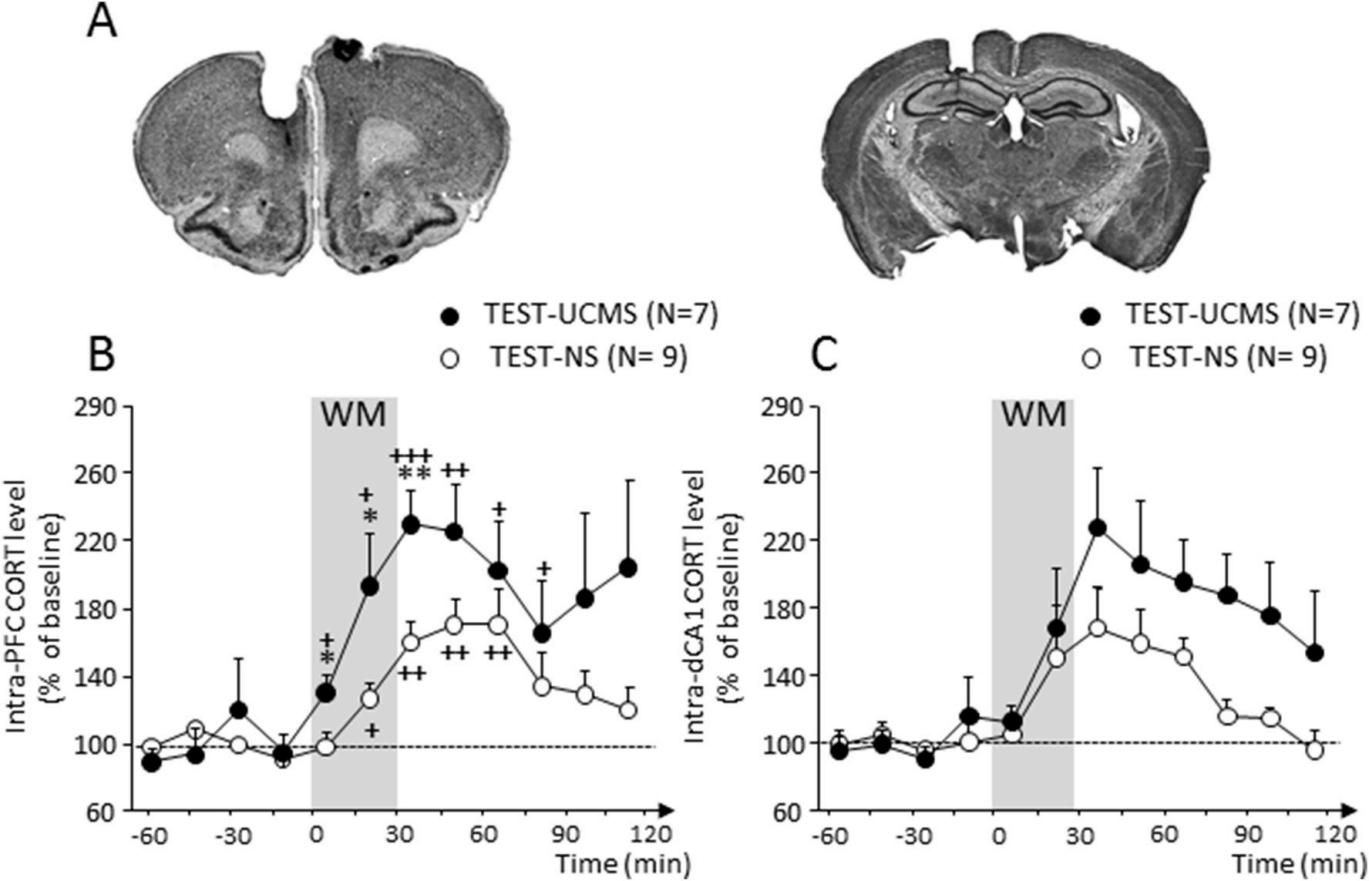
**Effects of *UCMS* exposure on corticosterone response in the PFC and dHPC.** (**A**) Histological controls of intra-PFC *(left)* and intra-dCA1 *(right)*microdialysis probe implantation. Coronal brain sections were stained with cresyl violet. **(B)** In the PFC, *UCMS* mice exhibited significantly greater corticosterone concentrations during WM testing (grey area, 0 and 15-min time-points) and at 30-min relative to the non-stressed (NS) mice. **(C)** In the dHPC, the time lag to produce *UCMS*-associated increase in corticosterone concentrations was longer. No significant *UCMS* effect was found in the dHPC. Results are expressed as percentage of mean baseline values. Bars represent means ± SEM. *P < 0.05 and **P < 0.01 relative to *TEST-NS*. ^+^P < 0.05, ^++^P < 0.01 and ^+++^P < 0.001 relative to baseline.

**Fig. 4:**
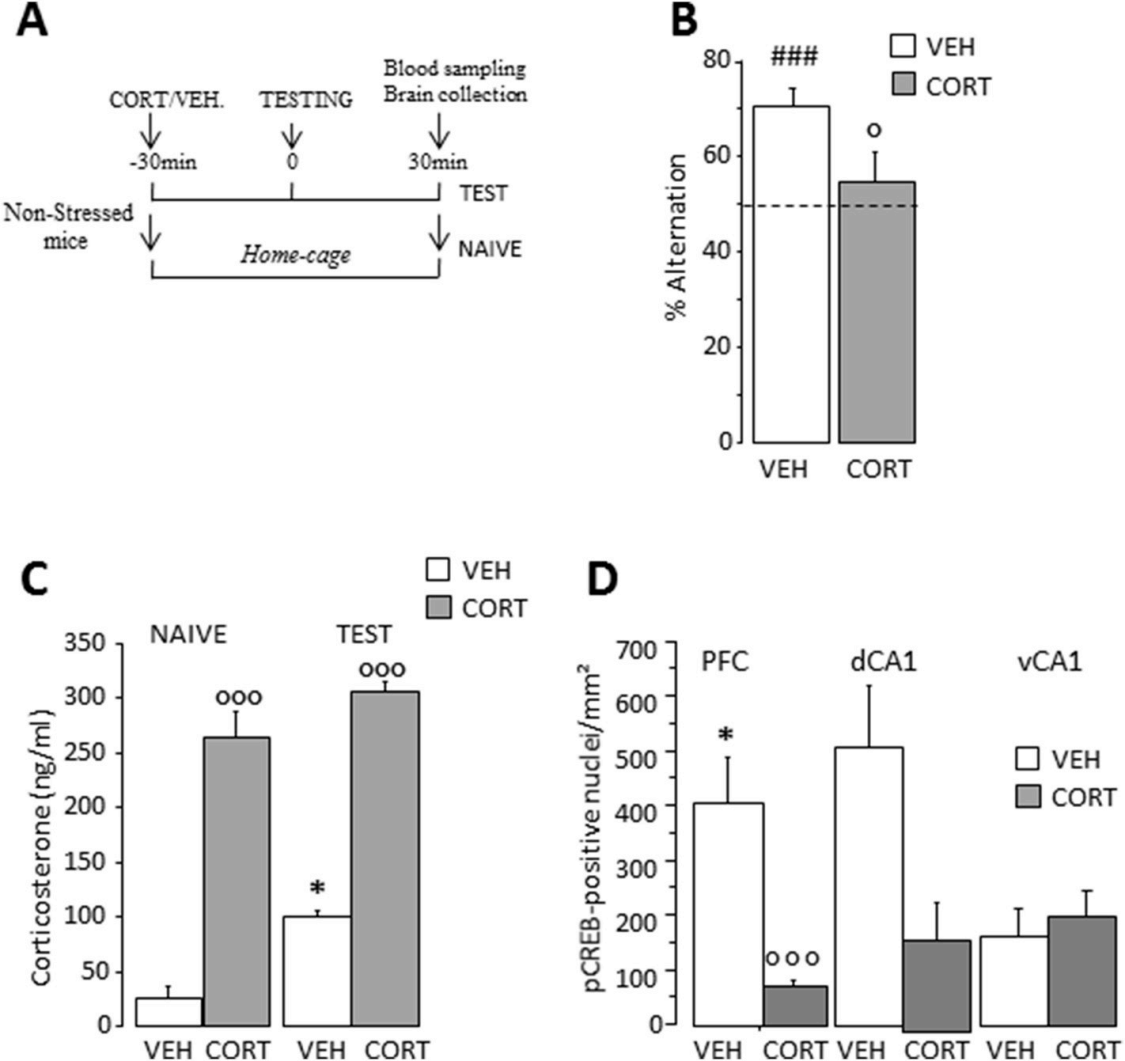
**Effects of pre-testing corticosterone injection in non-stressed mice. (A)** Plasma corticosterone concentrations and pCREB immunoreactivity were measured in non-stressed (NS) mice randomly assigned into *NAIVE* (CORT: N = 3; VEH: N = 3) or *TEST* (CORT: N = 7; VEH: N = 8). **(B)** Pre-testing CORT injection significantly reduced alternation rates in the NS mice. Dashed line represents chance level. **(C)** CORT injection significantly increased the plasma corticosterone concentration (ng/ml) under both *NAIVE* and TEST conditions. **(D)** CORT blocked the testing-associated increase in pCREB in the PFC and the dCA1. However, the effect was significant in the PFC only. Results are expressed as number of positive pCREB nuclei/mm^2^. Bars represent means ± SEM. ###P < 0.001 relative to chance level (50%). P < 0.05, P < 0.001 relative to vehicle. *P < 0.05 relative to *NAIVE*.

**Fig. 5:**
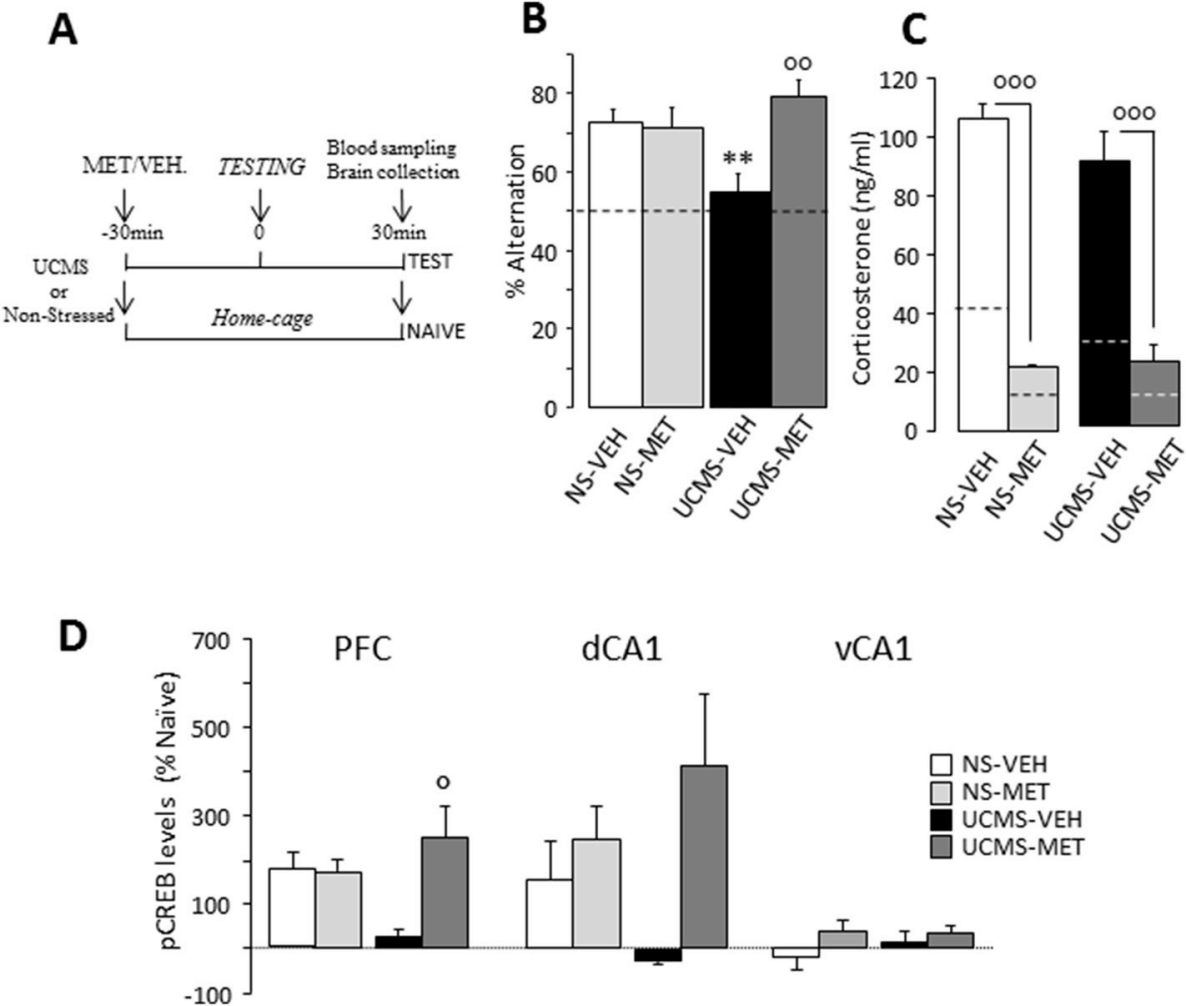
**Effects of metyrapone on WM performance, plasma corticosterone concentration and CREB phosphorylation in *UCMS* and NS mice. (A)** Experimental design: non-stressed (NS) and *UCMS* mice received metyrapone (MET) or vehicle (VEH) injection 30-min before sacrifice (*NAIVE*) or WM testing (TEST).Plasma corticosterone level and pCREB immunoreactivity were then measured 30-min after the beginning of WM testing and compared with respective naïve groups. **(B)** Metyrapone prevented the *UCMS*-induced WM deficits (as expressed as percentage of alternation rates). No drug effect was found in the NS group. Dashed line represents chance level. **(C)** Metyrapone blocked the effects of *UCMS* and testing on plasma corticosterone concentrations (ng/ml).Dashed lines represent corticosterone level in the four *NAIVE* groups.**(D)** Metyrapone prevented the effects of *UCMS* on changes in pCREB-positive neurons in the PFC and dCA1.Results are expressed as % variations of *NAÏVE*. Bars represent means ± SEM. **P < 0.01 relative to NS. °P < 0.05, P < 0.01 and P < 0.001 relative to VEH.

### 2.7 Statistical analysis

Statistical analyses were performed using the Statview 5.0 software^®^. Data were expressed as mean ± SEM. Behavioral and immunohistochemical data were analyzed using one- or two-way ANOVAs with *UCMS* exposure, Drugs and brain regions as independent factors. Results of multiple trials and time points were compared using two-way repeated measures ANOVA. Comparisons of WM performance with chance level were calculated with one-sample Student's t-test (with hypothesized mean-chance level = 50%). An analysis per block of three consecutive trials (Block A: trials 2 + 3+4; Block B: trials 5 + 6+7) were made in order to study proactive interference effects. Post-hoc Bonferroni/Dunnett's multiple comparisons analyses were performed when adequate. Significance was set at P ≤ 0.05.

## 3 Results

### 3.1 Effects of *UCMS* exposure on physical state and anxiety-like behavior

Measurements of the physical states (coat state score and body weight) were assessed weekly on mice randomly taken from the *UCMS* (n = 29) and non-stressed (n = 21) groups. As shown in http://www.sciencedirect.com.gate1.inist.fr/science/article/pii/S0166432812002124 Fig. 1B, the coat state scores of mice in each group did not differ between *UCMS* and non-stressed groups before the beginning of the *UCMS* procedure (week 0: both P > 0.1). A repeated measure ANOVA on data related to the coat state scores indicated a significant effect of *UCMS* (F_(1,48)_ = 61.03, P < 0.0001), a significant effect of time (F_(5,240)_ = 17.5; P < 0.0001) as well as a significant *UCMS* X time interaction (F_(5,240)_ = 14.81; P < 0.0001). Specifically, *UCMS* exposure produced a significant deterioration of the coat state score that began after three weeks of stress exposure (versus non-stressed: P < 0.01) and worsened until week 6 (versus *non*-stressed at weeks 4–6: P < 0.0001 for all comparisons). Similarly, *UCMS* induced a significant deterioration of the body weight during the 6 weeks of assessment as attested by a significant effect of time (F_(5,240)_ = 15.38; P < 0.0001; repeated ANOVA) and a significant time X *UCMS* interaction (F_(5,240)_ = 22.93; P < 0.0001; repeated ANOVA). As shown in Fig. 1C, only the non-stressed mice gained weight significantly (versus *weeks* 0-1; week 2: both P < 0.01; weeks 4–6: P < 0.001 for all comparisons).

After a 1-week resting period, the effects of *UCMS* on anxiety-like behavior in the elevated plus maze were examined on mice randomly taken out from each group (N = 13 each). As shown in Fig. 1D, the *UCMS* mice displayed significantly reduced time and distance spent exploring the open arm relative to non-stressed mice (0–10 min; time: F_(1,24)_ = 4.63; P = 0.04; distance: F_(1,24)_ = 5.5; P = 0.02; one-way ANOVA), indicating that *UCMS* exposure induced increased anxiety-like behavior. In contrast, total distance exploring the elevated plus maze was not statistically different across groups (F_(1, 24)_ = 0.4; NS; Fig. 1D _***right***_), indicating little influence of *UCMS* on general locomotor activity in this paradigm.

The hole-board exploration in the *UCMS* and non-stressed mice was tested 48 h after the elevated plus maze (Fig. 1E). Statistical analyses on the time spent inspecting the holes and number of holes visited during the two 0–5min and 5–10-min observation periods indicated that significant effects of *UCMS* exposure (F_(1,24)_ = 13.36; P < 0.0013 and F_(1,24)_ = 8.43; P < 0.0078; respectively), of observation periods (F_(1,24)_ = 9.9; P < 0.0044 and F_(1,24)_ = 5.44; P = 0.028, respectively) as well as a significant interaction between *UCMS* X observation periods for time (F_(1,24)_ = 4.21; P = 0.05). Importantly, further analyses indicated that, whereas the two groups did not differ during the first 5-min period (time: P = 0.37; number of holes P = 0.056), non-stressed mice displayed significantly greater total time exploring the holes and greater number of total holes visited over the 5–10 min period observation relative to the stressed mice (time: P < 0.001; number of holes: P < 0.01) and to non-stressed condition during 0–5min period (time: P < 0.001; number of holes: P < 0.05).

### 3.2 Effects of *UCMS* on WM performances, serum corticosterone level and levels of CREB phosphorylation in the PL subregion of the PFC and the dorsal CA1 (dCA1) of the dHPC

The remaining *UCMS* (N = 10) and non-stressed (N=8) mice were tested in a T-maze sequential alternation task to examine whether chronic stress exposure caused WM deficits during the test session with a 90-sec ITI (Fig. 2A). The ANOVA revealed a significant effect of *UCMS* (F_(1,16)_ = 11.31; P = 0.004) and a significant *UCMS* x block effect (F_(1,16)_ = 8.0; P = 0.01). Indeed, Fig. 2B shows that *UCMS* mice exhibited normal alternation rates on Block A (trials 2–4) but were impaired on Block B (trials 5–7) relative to non-stressed mice (Trials 5–7; p < 0.001). One sample *t*-test comparisons to chance level (50%) indicated that only non-stressed mice were able to alternate significantly above chance (non-stressed: t(7) = 5.58; P < 0.001; *UCMS*: t(9) = 0.61; P = 0.5). In contrast, *UCMS* did not affect mean choice latencies all along trials 1–7 (non-stressed: 21.1 ± 2.5 s; *UCMS*: 16.7 ± 1.6 s; F_(1,18)_ = 2.2; P > 0.1). Finally, both groups exhibited high alternation rates when tested on an ITI of 5-sec (Fig. 2B; trial 8), thus excluding altered locomotor activity impairments or altered motivation to alternate as causal factors of the deficits observed on Block B.

Plasma corticosterone levels were significantly increased after *UCMS* exposure (F_(1,27)_ = 17.18; P = 0.0003), independently of the baseline or TEST conditions (Fig. 2C). Both *TEST* groups displayed significantly greater levels of plasma corticosterone relative to baseline controls (both p < 0.001) and the level was significantly higher in *UCMS* relative to non-stressed mice (P < 0.01), indicating an additive effect of stress exposure and testing. Then, we examined whether brain region-specific alterations in pCREB co-occur with WM deficits in stressed mice. The numbers of pCREB-positive neurons were measured in three regions (PL subregion of the PFC, dCA1 and vCA1 regions) in mice sacrificed 30min after the beginning of WM testing or immediately after removal from their home-cage (naïve values) (Fig. 2D–E). Two-way ANOVAs conducted in each region showed a significant *TEST* effect in the PL (F_(1,27)_ = 5.2; P = 0.03) and in the dCA1 (F_(1,27)_ = 13.09; P = 0.0012) as well as a significant *TEST* X *UCMS* interaction in both regions (PL: F_(1,27)_ = 12.31; P = 0.0016; dCA1: F_(1,27)_ = 5.46; P = 0.027). Under naïve condition, the *UCMS* mice displayed significantly greater number of pCREB-positive neurons in the PL subregion of the PFC and the dCA1 (P < 0.05 and P < 0.01, respectively) relative to non-stressed mice. After WM testing, the non-stressed, but not the *UCMS*, mice displayed significantly greater number of pCREB-positive neurons in both structures compared to naïve values (both P < 0.001). In the vCA1, ANOVA revealed no significant *TEST X UCMS* interaction nor were there effects of *UCMS* or TEST.

### 3.3 *UCMS* led to significant elevation of corticosterone level in response to WM testing specifically in the PFC

The time-course evolution of corticosterone concentrations was examined 60-min before (basal level), during (0 to +30-min) and 90-min after WM testing in independent cohorts of mice which were implanted mice were implanted with microdialysis probes to measure CORT levels in the PFC or dHPC (non-stressed: N = 9; *UCMS*: N = 7) (Fig. 3A). As expected, *UCMS* exposure significantly reduced alternation rates on Block B with an ITI of 90-sec ec (*UCMS*: Trials 5–7: 53.6 ± 4.3%; non-stressed: 75.0 ± 3.6%,; P < 0.001). In contrast, all mice alternated similarly and significantly above chance level when tested on trial 8 with a 5-sec ITI (data not shown).

As shown in Fig. 3B–C, there was no significant *UCMS* effect on baseline corticosterone concentrations (i.e. mean ± SEM from 4 dialysates taken before behavioral testing) in the PFC (non-stressed: 0.244 ± 0.03 ng/ml and *UCMS*: 0.205 ± 0.07 ng/ml; P > 0.5) and in the dHPC (non-stressed: 0.196 ± 0.02 ng/ml and *UCMS*: 0.156 ± 0.02 ng/ml; P > 0.5). ANOVA's analyses performed on data from samples taken immediately to 120-min after the beginning of WM testing yielded a significant *UCMS* effect in the PFC (F_(1,98)_ = 4.8; p = 0.04) but not in the dHPC (F_(1,98)_ = 3.7; p = 0.07) along with a significant time effect in both structures (PFC: F_(7,98)_ = 4.3; p < 0.001; dHPC: F_(7,98)_ = 5.35; p < 0.001). In the PFC (Fig. 3B), WM testing elicited a significant increase of corticosterone in the *UCMS* mice compared to non-stressed mice (0–15min: p < 0.05; 30min: p < 0.01), which persisted in the post-testing samples. In contrast, in the dHPC (Fig. 3C), an elevation in corticosterone concentration only emerged in the 30–60min (non-stressed) and the 30–90min (stressed) post-testing time-points compared to baseline values. However, no significant *UCMS* effect was found in the dHPC (p > 0.10 in all analyses).

### 3.4 Pre-testing injection of corticosterone in non-stressed mice produced effects similar to *UCMS*

Based on the data presented above, the prolonged and sustained elevation of corticosterone concentration emerges as a key factor of *UCMS*-induced WM impairments. Thus, we then examined whether non-stressed mice receiving pre-testing injection of corticosterone (CORT) exhibited behavioral and molecular alterations similar to those observed in the *UCMS* animals (Fig. 4A). As shown in Fig. 4B, the total alternation performances of the two groups were significantly different (P < 0.05) and only non-stressed mice injected with vehicle (VEH) were able to alternate significantly above chance level (VEH: t(7) = 5; P < 0.001; CORT: t(6) = 0.79; P = 0.45).

Concerning plasma corticosterone levels, ANOVA indicated significant effects of Drug (F_(1,17)_ = 369; P < 0.0001) and TEST (F_(1,17)_ = 24.9; P < 0.0001), without Drug X TEST effect (F_(1,17)_ = 1.89; P > 0.1). Fig. 4C shows that the CORT- and VEH-injected groups significantly differed before and after WM testing (P < 0.001 for both conditions). In the *TEST* groups, the non-stressed mice injected with VEH, but not with CORT, had significantly greater plasma corticosterone levels relative to respective baseline controls (VEH: P < 0.0001 and CORT: P = 0.07).

Fig. 4D shows the effects of pre-testing CORT injection on pCREB in the PL subregion of the PFC, the dCA1 and the vCA1. In the Naive groups, no Drug effect was found, whatever the region examined (data not shown). In the *TEST* groups, two-way ANOVA yielded a significant effect of Drug (F_(1,13)_ = 8.62; P = 0.016), a significant Drug X Region interaction (F_(2,26)_ = 5.62; P < 0.01) but no effect of Region (F_(2,26)_ = 2.74; P = 0.08). Post-hoc analyses confirmed that non-stressed mice injected with CORT had significantly reduced pCREB levels in the PFC and the dCA1 but not in the vCA1 compared to the VEH-injected non-stressed mice (PFC: P = 0.0027; dCA1: P = 0.02; vCA1: P > 0.1).

### 3.5 Pre-testing injection of metyrapone prevented the *UCMS*-induced behavioral and molecular alterations

We next investigated whether the corticosterone synthesis inhibitor metyrapone (MET) administrated 30min before TEST could reverse all the effects of *UCMS* exposure (Fig. 5A). In non-stressed animals, WM performances were above chance level whatever the treatment considered (VEH: t(7) = 5.22; P = 0.0012; MET: t(7) = 5; P = 0.0016; Fig. 5B). The *UCMS* mice injected with MET alternated successfully (t(7) = 7; P < 0.001) in contrast to VEH-injected *UCMS* animals (t(6) = 1.0; NS), indicating that MET prevented WM impairments in stressed mice. Concerning plasma corticosterone concentration, the ANOVA analysis evidenced a significant Drug effect in the TEST groups (F_(1,27)_ = 68.38; P < 0.0001) but no significant *UCMS* nor interaction effects (both F_(1,24)_<1.0; both P=NS). Fig. 5C shows that pre-testing MET injection significantly reduced plasma corticosterone levels, independently of whether or not mice underwent *UCMS* exposure.

We next examined whether pre-testing injection of metyrapone can prevent the effect of *UCMS* on pCREB levels in the PL region of the PFC and the dCA1. Results were expressed as changes in percentage in pCREB levels relative to matched naïve mice remaining in their home-cage (Fig. 5D). In the PFC, there was a significant effect of Drug (F_(1.26)_ = 3.92,P = 0.058) and a significant Drug X *UCMS* interaction (F_(1.27)_ = 5.17, P = 0.031). As expected, VEH-injected *UCMS* mice showed significantly reduced pCREB in the PFC compared to non-stressed animals, independent of VEH or MET condition (P = 0.05 for both) (Fig. 5D_left_). In addition, prefrontal pCREB levels were significantly higher in the MET-injected *UCMS* mice compared to matched VEH-injected animals (P < 0.01) indicating that MET blocked the effects of stress on WM-related changes in pCREB in the PFC. In contrast, there were no main effects of *UCMS* or Drug, or interaction in the dCA1 (Fig. 5D_middle_) and vCA1 (Fig. 5D_right_).

## 4 Discussion

Our study shows that, when measured after one week resting period, the *UCMS* mice displayed WM deficits and enhanced plasma corticosterone response before and after WM testing. In addition, we found that *UCMS* mice displayed high basal pCREB level in the PFC and the dHPC and that WM testing did not elicit any further increase in pCREB. Microdialysis sampling of corticosterone concentration in the PFC revealed enhanced corticosterone response during WM testing selectively in the PFC in non-stressed mice which was potentiated and prolonged in *UCMS* mice. In contrast, a non-significant rise of corticosterone was observed in the dHPC after WM testing only, reaching a peak 30–60min (non-stressed) and 90–105 min (*UCMS*) post-testing time-points. Administration of corticosterone before WM testing to non-stressed mice produced behavioral and molecular alterations similar to those observed in *UCMS* animals whereas pre-testing injection of corticosterone synthesis inhibitor metyrapone reversed the enduring effects of chronic stress exposure. These findings indicate that changes in corticosterone concentrations in the PFC are a key factor of cognitive and molecular alterations induced by *UCMS*.

Two measures enable us to assess the impact of the *UCMS* procedure on the physical state of the animals: the coat sate and the evolution of body weight. Grooming is an important aspect of the rodent behavioral repertoire that is very sensitive to stress (Kalueff and Tuohimaa, 2004). Moreover, previous reports have shown such grooming reduction after a psychosocial stress (Kramer et al., 1999; Flugge et al., 2001). Thus, the reduction in coat quality as a function of the exposure to *UCMS* is used to quantify a depression-like state, as reported in several previous experiments in the same model of depression (Isingrini et al., 2010; Nollet et al., 2012; Surget et al., 2011). We have evaluated the grooming quality during the 6 weeks stress exposure and we found an abnormal appearance of the fur, which may be explained by a decrease of grooming behavior likely related to an increase in alertness at the expense of grooming activities (Ducottet et al., 2004; Ducottet and Belzung, 2004). We have also evidenced a significant decrease in weight gain in *UCMS* mice relative to control NS mice, although our procedure did not include any food and water deprivations, consistent with previous studies using animal models of depression (Griebel et al., 2002; Zhu et al., 2014). Moreover, consistent with previous findings of Zhu et al. (2014), our results demonstrate that *UCMS*-exposed mice displayed enhanced anxiety as measured by the decrease in the number of entries and the distance spent in open arms in the EPM test, in spite of the fact that the general locomotor activity did not differ between non-stressed and *UCMS* mice. Interestingly, *UCMS* also reduced exploratory activity in the second 5-min block of the exploratory session in the hole-board whereas exploratory activity was similar to that of controls in the 1st 5-min block. One possible explanation of the between-groups difference on the 5–10min block could be that non-stressed mice would habituate more rapidly than *UCMS* mice to the apparatus, which would enhance exploratory behavior in the 2nd 5-min block. Thus, the decrease of exploratory activity in *UCMS* mice would result from the maintenance of fear reactivity over the 2nd 5-min block rather than to an alteration of exploratory tendency *per se*. These overall data confirm that the chronic stress exposure induced an increase of anxiety and a deterioration of the physical state revealing a depression-like behavior in *UCMS*-exposed mice.

In parallel, we observed that *UCMS* pre-exposure leads to WM disturbance in a sequential alternation test. The low alternation rates observed in the *UCMS* mice were not attributable to decreased motivation to alternate nor to alteration of exploratory behavior during trial series, as performances were not compromised when the ITI was shortened from 90-sec to 5-s. Thus, the deficits observed in *UCMS* mice during WM testing resulted in an enhanced vulnerability to delay-dependent interference over a series. The findings that WM deficits were observed 7 days after the last day of *UCMS* exposure are consistent with evidence that prior exposure to chronic stress, or prolonged elevation of corticosterone, produces long-lasting behavioral deficits in mice and rats (Matthews and Robbins, 2003; Olausson et al., 2013). In line with other findings, clinical data indicated that chronic psychosocial stress selectively disrupts several executive PFC functions in human, including WM, selective attention and behavioral flexibility (Negrón-Oyarzo et al., 2016).

Dysfunction of the HPA axis is one of the most common disturbances reported in depression. Indeed, clinical observations show that most depressive patients exhibit a hyperactivity of the HPA axis, inducing a high GCs secretion and a disruption of the negative feedback loop on further GCs secretion (Holsboer, 2000). Thus, we attempted to determine whether *UCMS*-induced cognitive alterations are accompanied by abnormalities of the HPA axis functioning. In our study, *UCMS* exposure elicited an increase in plasma corticosterone concentration when measured at both baseline and 30min after the beginning of WM testing, which is consistent with other previous findings (Herman et al., 1995; Johnson and Yamamoto., 2009). This is also consistent with evidence showing that systemic administration of GCs or local glucocorticoid infusion in the PFC caused PFC dysfunction and WM impairments (Arnsten, 2009; Barsegyan et al., 2010). Together, the findings suggest that exposure to chronic or repeated stressful events, via disruption of the negative feedback modulation of the HPA axis and abnormal excessive levels of circulating corticosterone concentration, can have detrimental effects on PFC structure and function. Using microdialysis samples from freely-moving mice, we examined whether *UCMS*-induced WM deficits co-occur with alterations in the region-specific temporal evolution of local corticosterone in the PFC and the dHPC. Our results indicated that non-stressed mice display a marked increase of corticosterone rise during WM testing specifically in the PFC and that *UCMS* potentiates the prefrontal corticosterone response to testing. The origin of corticosterone measured in the PFC and dHPC is unclear. Corticosterone may originate from adrenal production, but an extra-adrenal origin cannot be excluded. Such an extra-adrenal production of corticosterone could explain the difference in the exaggerated rise of corticosterone concentrations over the different time-points after behavioral testing in *UCMS* mice. However, there is still a matter of debate as to whether extra-adrenal corticosteroids are of any physiological significance. This will depend on factors such as local concentration proximity to target cells and, possibly, to tissue-specific control mechanisms (Davies and MacKenzie, 2003).

In contrast, non-stressed mice displayed a more progressive, non-significant, rise of corticosterone in the dHPC that took place after WM testing. In addition, although hippocampal corticosterone rise was greater and lasted longer in *UCMS* mice, we found no evidence of a significant *UCMS* effect on corticosterone response in the dHPC. Such brain regional difference in the pattern of local corticosterone response could reflect either a different involvement of the PFC and the dHPC in processing the WM task, with an earlier involvement of the PFC as regards dHPC, or a different temporal sensitivity of these regions in response to behavioral stress. In agreement with the latter hypothesis, Caudal et al. (2014), showed that the subcellular trafficking of corticosteroid receptors display distinct temporal dynamics in different limbic regions after behavioral stress.

Studies in animals have indicated that under stressful conditions, high levels of catecholamines, noradrenaline and dopamine, compromise the functional PFC integrity and lead to WM impairment, at least in part via an activation of the cAMP/protein kinase A (PKA) signaling transduction cascade (Barsegyan et al., 2010; Jett and Morilak, 2013). Indeed, activation of cAMP/PKA cascade leads to phosphorylation of CREB at Ser133 and subsequent induction of its downstream target gene expression, including genes required for learning and memory processes (Kida and Serita, 2014). However, previous evidence indicates that activation of the cAMP/PKA/CREB cascade improves HPC-dependent memory processes but impairs PFC-dependent memory processes, and particularly WM (Barsegyan et al., 2010). Moreover, abnormally active basal cAMP-PKA signaling cascade can occlude normal PKA-dependent processes leading to cognitive impairments (Barsegyan et al., 2010; Giralt et al., 2011). In agreement, we found that UCMS mice displayed increased basal pCREB immunoreactivity in the PFC and the dHPC compared with non-stressed animals and that WM testing did not elicit further increases in pCREB in *UCMS* mice as it did in non-stressed ones. Supporting the view that abnormal activation of basal PKA-CREB cascade may be a key determinant of WM impairments in *UCMS* mice, our pharmacological data indicated that in *UCMS* mice, systemic administration of the corticosterone synthesis inhibitor metyrapone, prevents WM impairments and normalizes prefrontal pCREB activity in both basal and testing conditions. In contrast, markedly increased plasma corticosterone in non-stressed mice given a pre-testing injection of corticosterone elicited WM impairments along with significantly reduced pCREB in the PFC. Even though we did not measure directly the regional corticosterone concentrations in metyrapone-treated mice, these findings suggest that systemic administration of metyrapone in *UCMS* mice, by reducing the plasma corticosterone concentrations, could weaken or suppress the abnormal rise of corticosterone observed during testing in the PFC and thus, restore normal WM performance as well as prefrontal pCREB activity in *UCMS* mice. In support of this view, we have previously shown that a systemic metyrapone injection prevents the contextual memory retrieval impairment induced by a pre-test acute stress *via* the blockade of the stress-induced rise of corticosterone specifically in the dHPC (Tronche et al., 2010; Chauveau et al., 2010).

In summary, we showed in *UCMS* and non-stressed mice that the improvement or impairment of WM performance are closely related to changes of pCREB activity mainly in the PFC according to regional corticosterone concentrations. All together, our data shows that the abnormal regional increase of corticosterone concentrations mainly in the PFC emerges as a key factor of WM and neural activity dysfunctions in *UCMS*-treated animals.

## Declarations of interest

None.

## Fundings

This study was financially supported by the CNRS and by a grant from the “Fondation pour la Recherche en Alcoologie (FRA), France # 2017/02” attributed to DB and NM.
